# Confronting the Ethical Challenges of Big Data in Public Health

**DOI:** 10.1371/journal.pcbi.1004073

**Published:** 2015-02-09

**Authors:** Philip E. Bourne

**Affiliations:** Office of the Director, National Institutes of Health, Bethesda, Maryland, United States of America

“Ethical Challenges of Big Data in Public Health” by Vayena et al. is a timely paper for a number of reasons, all of which are concisely embodied within the title. Ethical challenges are not something we consider often enough in this or most biomedical research journals. Yet, such challenges increasingly confront us as private citizens providing health data and as researchers using these data for public health and research purposes.

Big data is an ill-defined term, yet there can be no denying that social media data (which are described in this article for use in digital disease detection [DDD]) cover a large and varied area, measured by both volume and velocity (rate of accumulation) and, as the authors describe, have the potential for important public health uses, but also have risks. And that, as described, is only one potential use of these data; there are many other uses barely introduced by the authors. In short the appeal of such data is huge, but so are the risks. The authors do an excellent job of characterizing the pluses and minuses of DDD. I encourage all of us to contemplate this article and the implications it masterfully elucidates.

The underlying message from Vayena et al. is clear: use of these data far outpaces the governance and due diligence of the ethical considerations that need to be addressed. Quoting the authors,

stigmatization of particular communities which may adversely affect individual members; and even the infringement of individual freedoms, such as the freedom of movement of an individual falsely identified as a carrier of a particular disease.…

the same problems associated with current disease detection and public health approaches are likely to be more pronounced with the advent of social media and big data. In fact, DDD will only exacerbate the challenges of preventing misuse of the data. The authors are to be commended for raising the profile of this important issue and getting us thinking.

Interestingly, the framework for addressing the issue has been around for a long time. It is embodied in Article 27 of the 1948 Universal Declaration of Human Rights, which declares it a human right “to share in scientific advancement and its benefits” (including to freely engage in responsible scientific inquiry) and at the same time have “the protection of the moral and material interests resulting from any scientific production of which [a person] is the author” [1].

More in a research context, this has recently been considered further by the global community and has been embodied in the Global Alliance for Genomic Health (GA4GH)’s work on a framework for responsible sharing of genomic and health-related data [2]. Governments and organizations worldwide have their own more in-depth versions. In the United States and within the National Institutes of Health (NIH), there is an update to the Common Rule underway [3], and a more extensive genomics data sharing plan was recently announced [4]. Further measures are under consideration for protecting the rights of the individual whilst ensuring that maximum access to patient data for appropriate research purposes is maintained to accelerate much needed cures and improvements to health care in general. As illustrated in this article, whether for purposes of research and/or public health, social media data require that further attention be paid. The authors are to be commended for raising the profile of these ethical issues.
